# Emotion Regulation Convoys: Individual and Age Differences in the Hierarchical Configuration of Emotion Regulation Behaviors in Everyday Life

**DOI:** 10.1007/s42761-023-00228-8

**Published:** 2023-12-16

**Authors:** Marissa A. DiGirolamo, Shevaun D. Neupert, Derek M. Isaacowitz

**Affiliations:** 1 Department of Psychology, Northeastern University, Boston, MA USA; 2 Department of Psychology, North Carolina State University, Raleigh, NC USA

**Keywords:** Experience-sampling method (ESM), Aging, Emotion regulation, Emotion regulation convoys, Tactics

## Abstract

A key limitation of studying emotion regulation behavior is that there is currently no way to describe individual differences in use across a range of tactics, which could lead to investigations of intraindividual changes over time or interindividual differences as a function of personality, age, culture, or psychopathology diagnosis. We, therefore, introduce *emotion regulation convoys*. This research tool provides a snapshot of the hierarchy of emotion regulation tactics an individual favors across everyday life situations and how effective they are at regulating moods. We present data from a 3-month measurement burst study of emotion regulation behavior in everyday life in a sample (*N* = 236) of younger (18–39), middle-aged (40–59), and older adults (60–87), focusing on how individuals’ convoys may vary in how much they include tactics that involve upregulating-positivity, downregulating-negativity, upregulating-negativity, as well as acceptance, and how these may be differentially effective. Among the most frequently used tactics (top tactics), older adults used a lower proportion of negativity-downregulating tactics than younger adults (*p* < .001), and younger adults’ mood was more negatively affected by these tactics than middle-aged and older adults. Overall, using positivity-upregulating as a top tactic also predicted better mood post-regulation. Older adults’ emotion regulation convoys may be made up of more effective tactics; in general, they reported more positive mood post-regulation than the other age groups. Convoys help us see emotion regulation as a hierarchical configuration of potentially effective behaviors, allowing us to test for between-group differences and within-person changes more precisely.

**Supplementary Information:**

The online version contains supplementary material available at 10.1007/s42761-023-00228-8.

One problem for researchers studying emotion regulation differences between (age, culture, diagnosis) groups is that strategies can be implemented by different *tactics* (Gross, 2015). Groups may differ in their tactic preference, tactic effectiveness, or both. For example, in prohedonic emotion regulation (trying to feel good/better), tactics of reappraisal may be focused on upregulating positive (positive reappraisal) or downregulating negative (detached reappraisal) aspects of the emotional situation; these valence-based tactics are shared across strategies (Livingstone & Isaacowitz, 2019). Perhaps one group favors tactics across multiple strategies that upregulate positivity. These tactics can include positive situation selection, focusing attention on positive, and positive reappraisal. Alternatively, another group may prefer tactics that downregulate negativity, such as avoiding negative situations, directing attention away from negative content, and detached reappraisal. When examining effectiveness, one group may benefit more from positivity-upregulation, whereas the other may benefit more from negativity-downregulation.

Studies of group differences in emotion regulation lack a tool to investigate the configuration of tactics used by an individual at a given time which could then be used to test for group differences or intraindividual changes over time. Critically, these tactics are hierarchically organized, because their *relative* frequency is necessary to give an accurate snapshot of individual tendencies. For example, two individuals may both show the same absolute frequency of positive attention deployment, but for one, it may be the most frequent tactic by far, whereas for another, it is less frequently used than many other tactics. Therefore, the relative importance of this tactic is greater for the first participant than for the second.

Here, we present *emotion regulation convoys* as a tool to provide hierarchically structured snapshots of an individual’s emotion regulation behavior at a particular time. In social gerontology, social convoys (Antonucci & Akiyama, 1987, p. 519) represent “a protective layer…who surround the individual and help in the successful negotiation of life’s challenges…Convoys are thought to be dynamic and lifelong in nature, that is, changing in some ways but remaining stable in others, across time and situations.” Social convoys are typically measured with circles (Kahn & Antonucci, 1981), in which individuals indicate their hierarchy of social partners: those so close they cannot imagine life without (inner circle), not as close (middle circle), and acquaintances (outer circle). Importantly, social partners in the inner circle are uniformly very close to the individual but may have no relationship to each other (i.e., parent, close work colleague).

A similar approach for emotion regulation convoys would include the hierarchy of most-used tactics: most frequently relied on across situations (could not function without: *inner circle*), not as critical but still important and regularly used (*middle circle*), and seldom used (*outer circle*). While social circles arise primarily from self-reports, emotion regulation convoys can be derived from experience sampling data by calculating the relative frequency of tactic use across situations for an individual. Like social convoys (Antonucci et al., 2014), emotion regulation convoys can capture both the quantity and quality of emotion regulation behavior by testing the hierarchically organized use of tactics and their effectiveness.

While emotion regulation convoys could help test any group differences or intraindividual changes (such as during or after psychotherapy), we consider them in the context of possible adult age differences. There are robust age differences in self-reported emotional experience; older adults typically report higher levels of emotional well-being than their younger counterparts (Carstensen et al., 2000; Charles & Carstensen, 2009; Mroczek & Kolarz, 1998; Neupert et al., 2007). This has led to claims that older adults are better at regulating emotions (Urry & Gross, 2010); however, evidence to date does not clearly support this assertion (Isaacowitz, 2022). Both lab studies and studies of everyday life have found essentially no age differences in strategy use or effectiveness (Eldesouky & English, 2018; Kunzmann et al., 2023; Livingstone & Isaacowitz, 2021). Without clear evidence or a consistent pattern for age differences in strategy use in prior work, it is impossible to understand or test theories of age-related change in emotion regulation. However, recent work has identified some age differences in specific tactic types across strategies (DiGirolamo et al., 2023; Livingstone & Isaacowitz, 2021), suggesting that examining emotion regulation at the tactic level is the most meaningful for assessing age differences. Specifically, positivity-upregulating tactics across strategy types are most frequently used regardless of age, but older adults may use them even more (Livingstone & Isaacowitz, 2021).

Although past studies testing only tactic frequencies (DiGirolamo et al., 2023; Livingstone & Isaacowitz, 2021) are suggestive that tactics are important for understanding age differences, testing frequencies alone cannot provide a precise snapshot of an individual’s emotion regulation behavior because it does not account for the hierarchical structure. It is impossible to test whether older adults rely on a different or more effective configuration without assessing individual differences in the *relative* reliance on some tactics versus others. Prior work has not directly examined this hierarchical aspect of tactic use and effectiveness.

The current paper fills this gap by addressing age differences in emotion regulation use and effectiveness in everyday life through the lens of hierarchical convoys. We focus on valence-based tactics because of recent work suggesting possible age differences in their use (Livingstone & Isaacowitz, 2021). Tactics are also at the most granular level of the hierarchical structure but are shared across strategy types, thus painting a more precise snapshot of emotion regulation behavior that can facilitate testing of intraindividual changes. Prior work has also suggested that individuals frequently report acceptance when asked how they regulate their emotions (i.e., Wolfe & Isaacowitz, 2022). Though acceptance is not included in the process model (Gross, 1998) and is not a valence-based tactic per se, accepting the situation or emotions resulting from it without trying to change them appears to be an important aspect of emotion regulation behavior to consider: some studies find that older adults use acceptance more than other age groups (Schirda et al., 2016; Wolfe & Isaacowitz, 2022; cf. Livingstone & Isaacowitz, 2021). Therefore, we also consider the relative use and effectiveness of acceptance.

While convoys have 3 circles (frequency bands), we chose to focus primarily on an individual’s most frequently used tactics (inner circle or “top tactics”) when examining effectiveness. If we are looking for emotion regulation behavior that helps contribute to older adults’ positive emotional experience, it makes sense to look first at the frequently used behaviors instead of those rarely used.

Thus, our central research questions are as follows: (1) Are there age-related differences in individual convoy makeup, specifically the most frequently used tactics (inner circle or “top tactics”) in the categories of positivity-upregulating, negativity-downregulating, negativity-upregulating, and acceptance? (2) How does top tactic use at the level of regulation instances (derived from individual convoys) predict effectiveness, and is this effect moderated by age? We hypothesize that there are age differences in emotion regulation behavior such that older adults will use more positivity-upregulating tactics as top tactics than younger adults, in line with previous experience sampling research on tactics (Livingstone & Isaacowitz, 2021), though we also acknowledge the possibility of similarities across age groups. For effectiveness, we hypothesize that using positivity-upregulating as top tactics at the emotion regulation instance level will predict higher self-reported affect post-regulation, but links between top tactic use and affect are expected to be moderated by age, such that positivity-upregulating tactic use will be more predictive of improved affect for older adults than younger. Thus, older adults who use positivity-upregulating as their top tactics may also report having higher post-regulation affect ratings than younger adults. We do not have any specific hypotheses on the middle-aged group due to limited previous research on middle-aged adults in the literature on emotional aging.

## Method

### Transparency and Openness

The results reported here are a subset of a project investigating the ways that people try to influence their emotions in everyday life (see https://osf.io/a6dnf for the full pre-registration). We report our sample size, all data exclusions, manipulations, and all measures in the study, and we follow JARS (Kazak, 2018). All datasets and analysis code generated and/or analyzed during the current study are available on the OSF repository at this private link. Research materials are available by emailing the corresponding author. Data were analyzed using R, version 4.0.4 (R Core Team, 2021) and the packages *psych*, Version 2.0.12 (Revelle, 2021), *lme4*, Version 1.1–26 (Bates et al., 2015), and *sjstats*, Version 0.18.1 (Lüdecke et al., 2021). The package *ggplot2*, version 3.3.3 (Wickham, 2016), was used for data visualization.

### Participants

This study was approved by the Northeastern University Institutional Review Board (IRB; #20–08-09). Participants were recruited from lab databases as well as through local advertisements and were considered eligible if they (a) were in the appropriate age range, (b) scored 21 points or higher on the Telephone Mini-Mental Status Exam (adapted from Newkirk et al., 2004), (c) did not have significant vision impairment, and (d) owned a smartphone or compatible device.

The final sample size consisted of 236 participants across three age groups: 82 young adults (ages 18-39, 66% female; 44% White, 29% African American, 15% Asian or Asian American, 6% unreported, 4% Other/Mixed, 2% American Indian), 77 middle-aged adults (ages 40-59; 58% female; 40% White, 40% African American, 8% Other/Mixed, 7% unreported, 5% Asian or Asian American), and 77 older adults (ages 60-87; 52% female; 65% White, 22% African American, 5% unreported, 4% Asian or Asian American, 4% Other/Mixed). Our pre-registered sample size was 75 young adults, 75 middle-aged adults, and 75 older adults based on a power analysis of 80% power to detect medium-sized age differences (53 participants would detect a minimum *η*2 of .07; .09 is considered medium). Our previous work using Bayes factors and a similar sample size (50 participants in each age group; Livingstone & Isaacowitz, 2021) has generally found positive support for age similarity when effect sizes are smaller than *η*2 = .03 (when effects are small or close to zero). Therefore, except for a small range of small effects (*η*2 between .03 and .07), a total sample of 159 could provide evidence supporting either age similarities or differences. A total of 245 participants completed the intake portion of the study (87 young adults, 80 middle-aged adults, 78 older adults). Of those 245 participants, 9 dropped out before the Experience Sampling Method (ESM) surveys. Of the remaining 236 participants who completed ESM surveys, 220 completed at least one burst and 198 completed more than one burst.

### Procedure

**Fig. 1:**
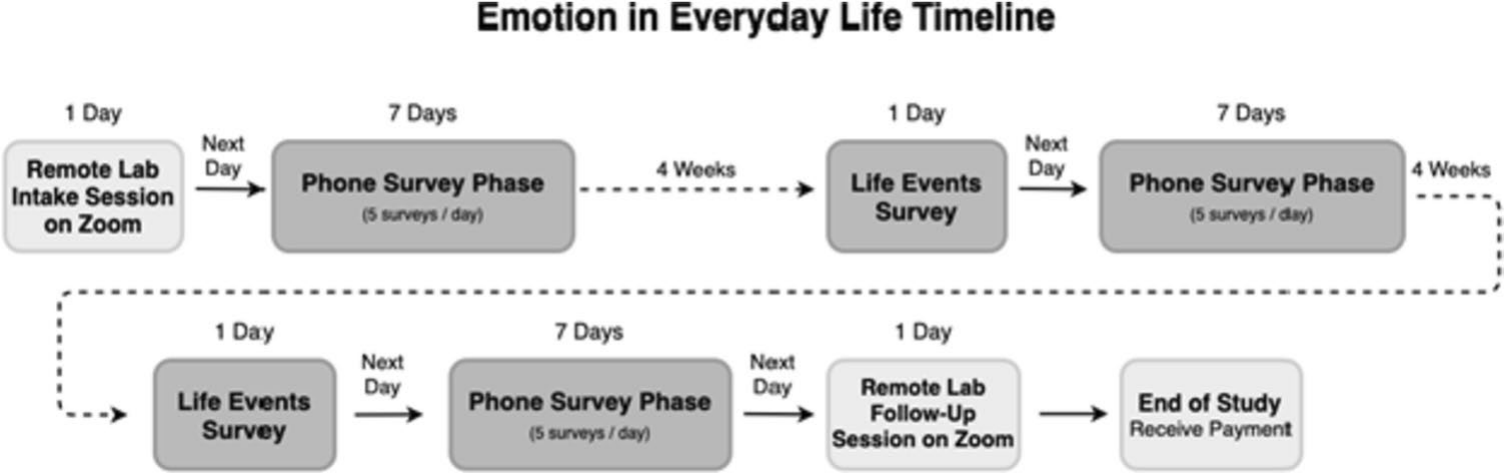
Study timeline indicating the 3 ESM bursts (phone survey phase) that were 1 month apart, and additional life events survey prior to phone survey phase (see Table 1 in Supplementary Materials for all measures)

This study was a 3 month-long longitudinal study using ESM. During this time, participants first completed an intake session over a HIPAA-compliant version of Zoom, three ESM measurement bursts of 7 days each, and then a final debrief session over the same HIPAA-compliant version of Zoom (see Fig. 1). An ESM mobile app was used (mEMA; ILUMIVU, 2009) which prompted participants to complete a survey five times a day for 7 days in a row. Prompts were delivered via both sound and text notifications. Participants chose a 12-h time frame, in which the surveys would be sent 5 times, randomly within 80-min windows spaced 30 min apart. They were asked to respond to survey notifications within 15 min, but after 1 h, the prompts would become unavailable in order to limit a backlog of surveys, duplication, or correcting of previous surveys. Participants completed three bursts total, with 4 weeks in between each burst.

“Bursts” as a measurement were outlined in the study’s design; however, due to technical issues, some participants re-started or delayed their initiation of a burst leading to too many observations or too much/too little time between bursts. Therefore, we calculated bursts based on the number of weeks passed since the last survey and manually adjusted individual instances that were still in the incorrect burst due to an interruption in the data collection. To preserve as much data as possible, we kept all those participants who had at least one burst with 13 or more instances completed.^1^ Table 1 summarizes details regarding participants included in this study by age group, including the percentages of participants who completed all of the bursts within each age group. At the intake session, participants gave their informed consent after receiving a thorough explanation of the study and study measures. Participants completed an in-depth training over Zoom in which survey questions and relevant definitions of emotion regulation strategies and tactics were explained in detail. First, researchers introduced participants to a 15-min animated video containing detailed explanations of what emotions are, how we influence our emotions, and the different emotion regulation strategies and tactics we may use in everyday life situations. Researchers were instructed to pay attention to the participants for any confusion and to pause the video if they indicated they had questions. Then, participants were asked to describe with a personal example when they have used the regulation strategies described in the video to change their emotions. Researchers corrected participants as they worked through the example and ensured they were comfortable with the idea of strategies and tactics, recording to what extent they understood and completed the training. The ESM app was installed, and participants were shown a demo version of the app and encouraged to ask for clarification. In the Debrief session, participants completed a series of other self-report measures (see Table 1 in Supplementary Materials for a complete list).

**Table 1:** Descriptive statistics for age groups, including percentage of completed bursts

Age group	*N*	% of sample	% of total sample that completed all bursts	# observations Median (min–max)	Mean Age	SD Age	Min Age	Max Age	% Male	% Female
Younger age (YA)	82	34	31	90 (15–105)	28.4	6.0	18	39	34	66
Middle age (MA)	77	33	29	91 (20–106)	50.2	6.0	40	59	39	61
Older age (OA)	77	33	31	94 (13–108)	68.7	6.1	60	87	45	55
Total	236	100	91	92 (13–108)						

To maximize completion rates, participants were paid a baseline amount ($40) for completing intake sessions and $1 for every survey completed on the ESM app, plus a bonus of $25 if they completed at least 75% of the survey prompts. If participants completed at least 90% of the survey prompts, they received a full payment of $200. In our past experience sampling study (Livingstone & Isaacowitz, 2021), this incentive structure motivated participants to complete the majority of surveys. In addition, participants received personalized feedback about their typical tendencies at the follow-up sessions (see Fig. 1 for a study outline), which they had found interesting and motivating in the past (Livingstone & Isaacowitz, 2021).

### Measures

For the measurement of all ESM variables, items were presented to participants in a fixed order. Participants received the same questions for every notification during the ESM survey bursts, and these questions changed based on branching that occurred from participant responses. Our analyses focused on the emotion regulation and self-reported mood measures explained below (see Supplemental Materials for descriptions of the additional measures included in the ESM survey).

#### Emotion Regulation Tactics

To target emotion regulation use, participants were asked, “Since the last survey, have you tried to do anything to influence your feelings?” where distinct items followed based on either a *yes* or *no* response (coded as 1 = yes, 0 = no). If participants answered *yes*, they were then asked about whether they used specific emotion regulation tactics (for the purpose of our analyses, we only focused on instances in which participants said “yes” to this question). We defined 17 unique emotion regulation tactics (see Table 2 for a full list of descriptions), broken down from five general families of emotion regulation strategies defined by the Gross (1998) process model (*situation selection, situation modification, attentional deployment, cognitive change/reappraisal, response modulation*). We also chose to explore two different forms of acceptance—*emotional* and *situational acceptance*.

**Table 2:** Emotion regulation strategy types broken down into tactics and tactic categories

Strategy	Tactic	Tactic category
Situation selection	Enter or seek out positive situations	
Situation modification	Change a situation to make it more positive	
Attentional deployment	Pay attention to positive aspects of a situation	Positivity-upregulating
Cognitive change/reappraisal	Think about the positive aspects or consequences of the situation	
Response modification	Put on a smile even if you felt negative	
Situation selection	Avoid or leave negative situations	
Situation modification	Change a situation to make it less negative	
Attentional deployment	Ignore or distract yourself from negative aspects of a situation	Negativity-downregulating
Cognitive change/reappraisal	Distance yourself or analyze the situation objectively	
Response modification	Hide the expression of emotion you were feeling	
Situation selection	Enter or seek out negative situations	
Situation modification	Change a situation to make it more negative	
Attentional deployment	Pay attention to the negative aspects of a situation	Negativity-upregulating
Cognitive change/reappraisal	Think about the negative aspects or consequences of the situation	
Response modification	Intentionally express or exaggerate expressions	
Emotional acceptance	Accept current emotions at any point	Acceptance
Situational acceptance	Accept the current situation at any point	

Each of the 17 tactics was presented as individual items in the ESM survey, with branching logic to account for valence and intention of the action. For example, if a participant responded *yes* to the question “did you select to enter or avoid a situation to influence your feelings?”, they were asked what specific action they took during selection (avoid or leave negative situations, enter or seek out positive situations, enter or seek out negative situations). If the participant responded *no* to the question “did you select to enter or avoid a situation to influence your feelings?”, the survey simply moved forward to asking if they used a different emotion regulation tactic. In order to make the convoys easier to parse, we grouped similar tactics from across different strategies into four tactic types: positivity-upregulation, negativity-downregulation, negativity-upregulation, and acceptance (tactics categories were drawn from Livingstone & Isaacowitz, 2021; see also Livingstone & Isaacowitz, 2016, Wolfe et al., 2022 for further discussion and studies using similar tactic categorization).

#### Pre- and Post-Regulation Self-Reported Affect

In order to measure the perceived effectiveness of tactic use, we asked participants to indicate how they felt before and after their regulation attempt using a Likert scale from 1 (“very negative”) to 7 (“very positive”; Luhmann et al., 2020). For example, if a participant indicated that they tried to change their emotions in any way (regulation attempt), they were then asked, “Before trying to change your emotions, how did you feel?” and “After trying to change your emotions, how did you feel?”.

### Data Analysis

For the purpose of our analyses, we only focused on regulation instances or instances in which participants indicated that they had tried to change their feelings. Overall, only 27% of all instances were reported as regulation instances. Additionally, all regulation instances resulted in higher, more positive affect for all age groups from pre- to post-regulation affect, indicating that participants’ reported regulation attempts were successful at changing their feelings.

#### Emotion Regulation Convoys

We used tactic frequency during the ESM period to calculate individualized emotion regulation convoys for each participant. All of these decisions regarding formation of the convoys were pre-registered, and we focused on a person-based approach when calculating the convoys. Each convoy consisted of three tertiles per individual: an inner circle representing the most frequently used tactics (or “top tactics”), a middle circle, and an outer circle representing the least frequently used tactics. We used a person-based approach to derive the convoys due to the individualized nature of this method, in which we first calculated the sum of the 17 tactics used within individuals and within each burst for each regulation instance (therefore, we had final sums for each tactic). If participants did not use one or more of the 17 tactics, indicated by a frequency of 0, the tactic was excluded from their convoy. Emotion regulation percentages were then calculated by dividing the total frequency of each tactic by the total number of regulation instances (only counted as a regulation instance when they used at least one of the 17 tactics) within each burst for each individual. It is important to note that participants could report using multiple tactics in a given regulation instance. For example, if a participant reported using the “Situation Selection Seek Positive” tactic a total of 14 times within the first burst, and the total number of times this participant reported regulating their emotions (and used any of the 17 tactics) was 20, the percentage for “Situation Selection Seek Positive” in burst 1 for this participant would be 70%.

With these calculations, we obtained frequencies for every single tactic within bursts for each individual. The frequencies of all the tactics for each participant were then ranked from the most frequently used to the least frequently used. Considering each individual could report using different numbers of tactics, we used this rank order to create tertile “bands” in which we identified the location of the 67th and 33rd percentile, indicating upper and lower cut-point numbers specific to each individual. The upper and lower cut-point numbers were determined only for the first burst and then used in later bursts. Tactic percentages that were greater than or equal to the upper cutoff number (the top 1/3 of tactics used) were placed in the participant’s inner circle; those less than or equal to the lower predetermined cutoff number (the bottom 1/3 of tactics used) were be placed in the participant’s outer circle, and percentages between the upper cutoff number (less than) and the lower cutoff number (greater than or equal to) were placed in the participant’s middle circle.

With this approach, two individuals may have the same frequency of one tactic, but they may not have these tactics within the same tertile band since this depends upon the frequency of all other tactics an individual uses. This method allows convoys to vary by individual, capturing the unique hierarchical configuration of tactics an individual uses at one point in time.

##### Tactic Categories

After calculating which tactics would be placed within each circle based on participant tertiles, we then created 4 new variables based on specific categories: positivity-upregulating, negativity-downregulating, negativity-upregulating, or acceptance-based (see Table 2).^2^ We used dummy coding to place each tactic into a specific category. For example, if a tactic was in the positivity-upregulating category, this variable would be coded as “1,” with all other categories coded as “0.” For each participant, we then aggregated the frequency of tactics used in each category by circles and bursts to create a percentage. For example, if a participant had a score of .20 for positivity-upregulating within burst 1 and the inner circle, this indicated that for burst 1, 20% of all the tactics in this participant’s inner circle were in the positivity-upregulating category. These percentages were then used in an ANOVA and pairwise comparison tests examining the age differences in percentages of tactics within each category.

For effectiveness analyses and the multilevel models, we specifically focused on the inner circle or the top tactics each participant used.^3^ Before analysis, dummy variables were created for whether a participant used at least one of the top tactics in general. For example, in a given regulation instance, if participants used at least one tactic that was within their inner circle convoy, the “top tactic use” variable was coded as “1” (otherwise “0”). Therefore, the results for this specific variable would be in reference to using a top tactic versus not using a top tactic. The “top tactic” variable was further broken down into whether the top tactic was in the Pos Up, Neg Down, Neg Up, or Acceptance category. For example, in a given regulation instance, if the use of a top tactic was also positivity-upregulating, then the positivity-upregulating variable (Pos Up) would be coded as “1” (otherwise “0”). Therefore, the “Pos up” variable referred to whether a tactic was a top tactic and also in the positivity-upregulating category versus any other category.

We checked the distribution of these categories and found that the negativity-upregulating category was positively skewed and resulted in significantly lower instances of being a top tactic (3.9%). Therefore, we opted to include only the other 3 tactic categories in the multilevel models.

## Results

### Age Effects on Between-Person Emotion Regulation Tactic Use and Self-Reported Pre- and Post-Regulation Affect

We calculated person-level mean scores for each measure during regulation instances only (self-reported pre- and post-regulation affect, 17 individual emotion regulation tactics, and additional “other” category; see Table 3 for descriptive statistics). Person-level mean scores for emotion regulation referred to the percentage that each individual chose a particular individual tactic over the entire course of the study (regulation instances only). For self-reported affect, the scores represent the average level of emotion participants reported experiencing pre- and post-regulation over the study duration. We conducted a series of ANOVAs and pairwise comparison tests to examine the age differences among person-level measures.

**Table 3:** Descriptive statistics (regulation instances only) for between-person measures by age group

	YA		MA		OA	
Items	*M*	*SD*	*M*	*SD*	*M*	*SD*
Affect components						
Self-reported pre-regulation affect	3.99	1.23	4.44	1.37	4.28	1.23
Self-reported post-regulation affect	5.10^c^	0.99	5.20	0.92	5.49^a^	0.85
Overall situation selection	0.52^bc^	0.28	0.64^ac^	0.30	0.74^ab^	0.24
Avoid or leave negative situations	0.20	0.24	0.26^c^	0.26	0.16^b^	0.18
Enter or seek out positive situations	0.35^c^	0.26	0.42^c^	0.31	0.61^ab^	0.27
Enter or seek out negative situations	0.04	0.09	0.03	0.08	0.03	0.05
Overall situation modification	0.65^c^	0.25	0.72	0.25	0.79^a^	0.22
Change a situation to make it less negative	0.32	0.28	0.31	0.26	0.26	0.22
Change a situation to make it more positive	0.42^c^	0.26	0.49	0.28	0.60^a^	0.28
Change a situation to make it more negative	0.04	0.08	0.03	0.06	0.02	0.03
Overall attentional deployment	0.58^bc^	0.27	0.71^a^	0.27	0.72^a^	0.25
Ignore or distract yourself from negative aspects of a situation	0.31	0.26	0.29	0.24	0.25	0.22
Pay attention to positive aspects of a situation	0.34^bc^	0.25	0.47^a^	0.29	0.52^a^	0.27
Pay attention to the negative aspects of a situation	0.04	0.08	0.04	0.10	0.03	0.07
Overall cognitive change/reappraisal	0.54	0.30	0.65	0.28	0.60	0.29
Distance yourself or analyze the situation objectively	0.25	0.25	0.24	0.22	0.21	0.20
Think about the positive aspects or consequences of the situation	0.35	0.26	0.43	0.29	0.42	0.29
Think about the negative aspects or consequences of the situation	0.06	0.12	0.05	0.11	0.05	0.09
Overall response modification	0.34	0.29	0.45	0.34	0.40	0.30
Hide the expression of emotion you were feeling	0.16	0.23	0.13	0.17	0.12	0.15
Put on a smile even if you felt negative	0.19	0.21	0.29	0.31	0.23	0.25
Intentionally express or exaggerate expressions	0.06	0.09	0.09	0.14	0.09	0.13
Overall acceptance	0.68^bc^	0.31	0.79^a^	0.23	0.80^a^	0.26
Accept current emotions at any point	0.58^c^	0.35	0.66	0.28	0.72^a^	0.29
Accept the current situation at any point	0.64	0.32	0.74	0.26	0.72	0.29
Other category	0.13	0.23	0.17	0.24	0.22	0.23

^a^ Significantly different from YA, ^b^ Significantly different from MA, ^c^ Significantly different from OA. Participants can select more than one tactic

The ANOVA revealed significant differences among age groups for individual tactics. For the individual tactics, older adults used enter or seeking out positive situations more than both middle-aged and younger adults, avoiding or leaving negative situations less than middle-aged adults, and changing the situation to be positive, attention to positive aspects, and accepting emotions more than younger adults. Middle-aged adults also used attention to positive aspects more than younger adults (all *p*’s < .03).

There were also significant age differences in self-reported post-regulation affect. Older adults reported significantly higher post-regulation affect than younger adults (*p* < .04), while there was no significant difference between the middle-aged and older or younger adults. Pre-regulation affect did not significantly differ between age groups (see Table 3).

### Age and Burst Effects on Overall Convoy Makeup

**Fig. 2:**
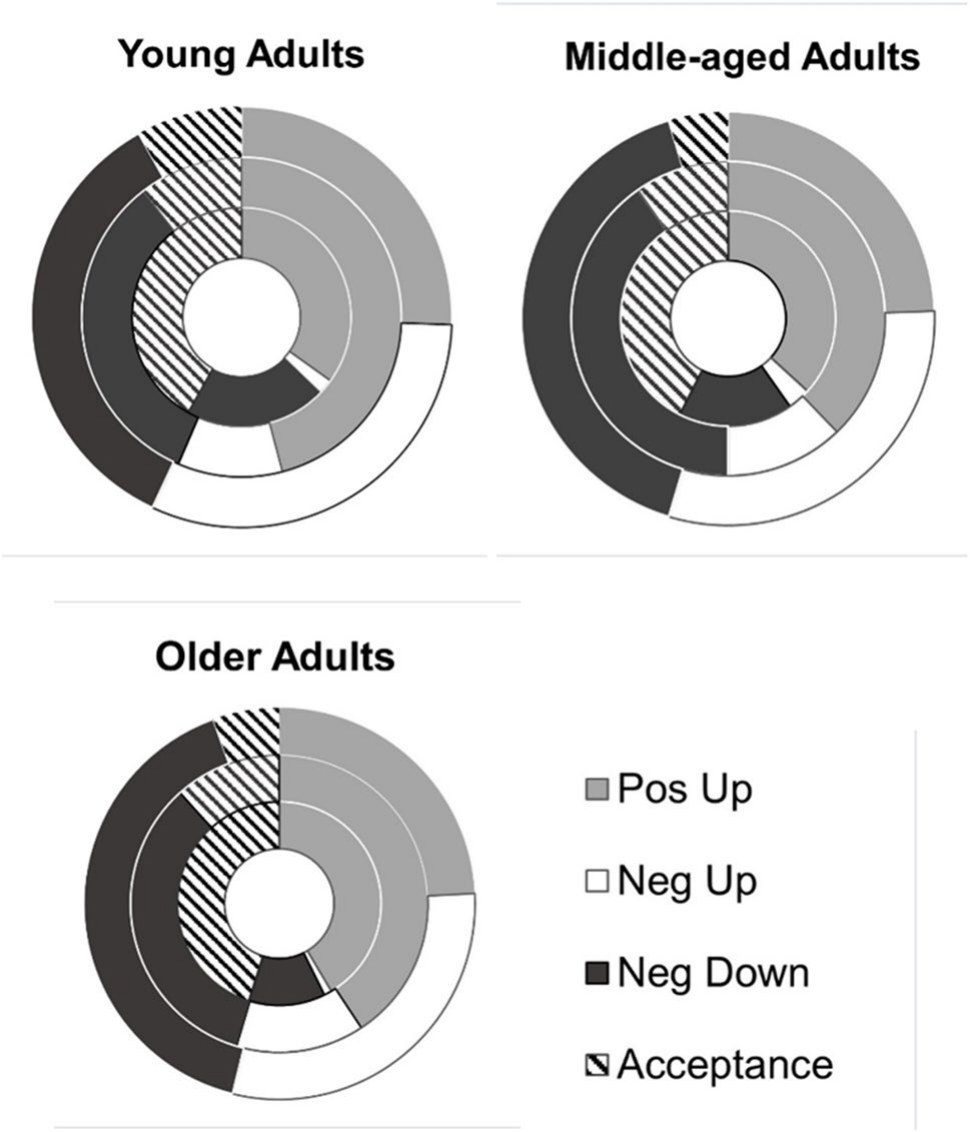
Tactic percentages split by age group, category type, and circle (inner, middle, outer) aggregated across bursts

We ran a 3 (age group: younger, middle-aged, older adults) × 4 (tactic: positive up, negative up, negative down, acceptance) × 3 (circle: inner, middle, outer) × 3 (burst: 1, 2, 3) ANOVA to investigate differences in the percentages of tactics that were positivity-upregulating, negativity-downregulating, negativity-upregulating, or acceptance-based among all convoys and bursts (see (Fig. 2) for percentages aggregated across bursts). This analysis helped us to determine whether there were age differences in the percentage of tactics used across and within convoys and the variation in percentages of tactics across bursts. These analyses also inform our choice to focus on top tactic use and whether they predict post-regulation affect ratings.

0The ANOVA revealed that burst was not a significant predictor, *F*(2,5212) = 0.10, *p* = .90, *η*^2^_*p*_ = .00, 95% CI [.00, .00]; therefore, there was no significant variation in the percentage of tactics used between bursts. The percentage of tactics used varied by category, *F*(3,5212) = 136.53, *p* < .001, *η*^2^_*p*_ = .07, 95% CI [.06, .09]. In general, participants used the highest percentage of tactics in the Pos Up category (*M* = 0.30, *SD* = 0.26), followed by Neg Down (*M* = 0.26, *SD* = 0.25), Acceptance (*M* = 0.17, *SD* = 0.20), and then Neg Up (*M* = 0.13, *SD* = 0.18; all *p*’s < .001). There was a significant convoy × category interaction, *F*(6,5212) = 167.16, *p* < .001, *η*^2^_*p*_ = .16, 95% CI [.14, .18], where for Pos Up-based tactics, the middle circle (*M* = 0.35, *SD* = 0.28) had significantly higher percentages than the inner and outer circle; for Neg Down, the outer circle (*M* = 0.34, *SD* = 0.29) had a significantly higher percentage than the inner circle; and for Acceptance tactics, the inner circle had a significantly higher percentage compared to all other circles (*M* = 0.37, *SD* = 0.27; all *p*’s < .005; see right side of Table 4).

**Table 4:** Means and standard deviations for percentage of tactics within the convoys as a function of a 4 (categories: positivity upregulating, negativity upregulating, negativity downregulating, acceptance) × 3 (age group: young—YA, middle—MA, older—OA) × 3 (circle: inner, middle, outer) × 3 (burst: 1, 2, 3) design

			Age Group							
			YA		MA		OA		Overall	
			*M*	*SD*	*M*	*SD*	*M*	*SD*	*M*	*SD*
Category	Convoy	Burst								
Positivity-upregulating	Inner	1	0.31	0.24	0.34	0.22	0.36	0.23		
		2	0.30	0.27	0.28	0.22	0.31	0.28		
		3	0.28	0.26	0.36	0.26	0.34	0.23		
		Avg	0.30^b^	0.26	0.32^c^	0.23	0.34^c^	0.25	0.32^c^	0.25
	Middle	1	0.37	0.23	0.36	0.24	0.33	0.24		
		2	0.36	0.30	0.36	0.30	0.39	0.33		
		3	0.44^^^	0.31	0.22^+^	0.28	0.32	0.28		
		Avg	0.39^ac^	0.28	0.32^c^	0.27	0.35^c^	0.28	0.35^c^	0.28
	Outer	1	0.23	0.32	0.20	0.26	0.19	0.25		
		2	0.24	0.27	0.23	0.22	0.21	0.27		
		3	0.20	0.24	0.19	0.19	0.26	0.27		
		Avg	0.22^b^	0.28	0.21^ab^	0.23	0.22^ab^	0.26	0.22^ab^	0.26
	Overall		0.30	0.27	0.28	0.24	0.30	0.26	0.30	0.26
Negativity-upregulating	Inner	1	0.01	0.05	0.03	0.10	0.01	0.05		
		2	0.03	0.08	0.04	0.11	0.01	0.05		
		3	0.03	0.09	0.03	0.15	0.01	0.04		
		Avg	0.02^bc^	0.07	0.03^bc^	0.12	0.01^bc^	0.05	0.02^bc^	0.08
	Middle	1	0.12	0.17	0.12	0.19	0.13	0.18		
		2	0.08	0.20	0.06	0.12	0.10	0.16		
		3	0.09	0.20	0.12	0.24	0.12	0.25		
		Avg	0.10^ac^	0.19	0.10^ac^	0.18	0.12^ac^	0.20	0.10^ac^	0.19
	Outer	1	0.34	0.30	0.26	0.27	0.29	0.29		
		2	0.25	0.27	0.22	0.28	0.24	0.26		
		3	0.23	0.23	0.31	0.27	0.27	0.25		
		Avg	0.27^ab^	0.27	0.26^ab^	0.27	0.27^ab^	0.27	0.27^ab^	0.27
	Overall		0.13	0.18	0.13	0.19	0.13	0.17	0.13	0.18
Negativity-downregulating	Inner	1	0.20^*^	0.22	0.13	0.19	0.10^+^	0.16		
		2	0.16^*^	0.18	0.18^*^	0.26	0.08^+^^	0.16		
		3	0.17	0.24	0.15	0.16	0.11	0.19		
		Avg	0.18^bc*^	0.21	0.15^bc*^	0.20	0.10^bc+^^	0.17	0.14^bc^	0.20
	Middle	1	0.30	0.23	0.30	0.22	0.28	0.21		
		2	0.29	0.29	0.32	0.26	0.26	0.29		
		3	0.26	0.28	0.38	0.32	0.32	0.28		
		Avg	0.28^a^	0.27	0.33^a^	0.27	0.29^ac^	0.26	0.30^a^	0.26
	Outer	1	0.27	0.28	0.37	0.31	0.37	0.29		
		2	0.30	0.28	0.37	0.32	0.40	0.32		
		3	0.33	0.24	0.31	0.26	0.35	0.30		
		Avg	0.30^a^	0.27	0.35^a^	0.29	0.37^ab^	0.30	0.34^a^	0.29
	Overall		0.25	0.25	0.28	0.25	0.25	0.24	0.26	0.25
Acceptance	Inner	1	0.35	0.25	0.38	0.21	0.37	0.23		
		2	0.33	0.30	0.39	0.28	0.40	0.29		
		3	0.39	0.33	0.34	0.25	0.36	0.26		
		Avg	0.36^bc^	0.29	0.37^bc^	0.25	0.37^bc^	0.26	0.37^bc^	0.27
	Middle	1	0.07	0.14	0.07	0.17	0.10	0.18		
		2	0.13	0.24	0.08	0.16	0.11	0.23		
		3	0.09	0.16	0.10	0.2	0.09	0.15		
		Avg	0.09^a^	0.18	0.08^a^	0.18	0.10^ac^	0.18	0.09^ac^	0.18
	Outer	1	0.07	0.20	0.02	0.10	0.04	0.12		
		2	0.06	0.15	0.05	0.16	0.04	0.16		
		3	0.09	0.22	0.04	0.12	0.07	0.12		
		Avg	0.07^a^	0.19	0.04^a^	0.12	0.05^ab^	0.14	0.05^ab^	0.15
	Overall		0.17	0.22	0.16	0.18	0.17	0.19	0.17	0.20

*M* and *SD* represent mean and standard deviation, respectively. Between convoy comparisons (within age groups): ^a^ Significantly different from inner circle, ^b^ Significantly different from middle circle, ^c^ Significantly different from outer circle, within convoy comparisons (across age groups): ^+^ Significantly different from YA, ^^^ Significantly different from MA, ^*^ Significantly different from OA (all *p*’s < .05)

**Fig. 3:**
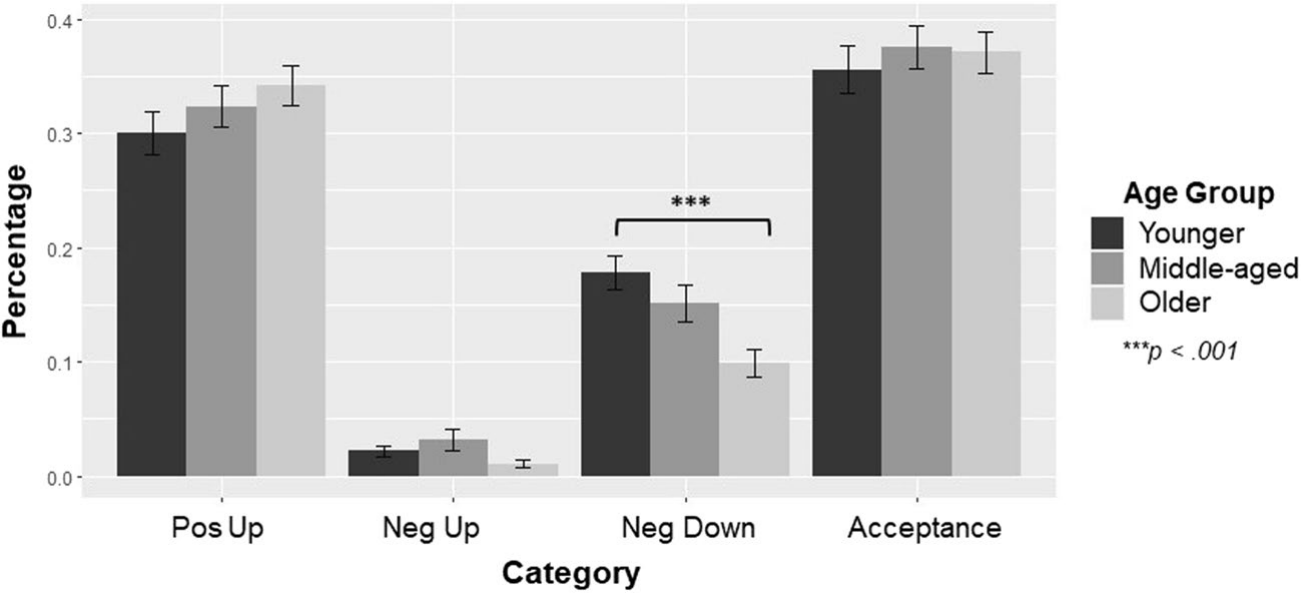
Convoy percentages split by age group and tactic category type for the inner circle convoy

There was also a significant convoy (inner, middle, outer) × age (younger, middle, older) × tactic category (Pos Up, Neg Down, Neg Up, Acceptance) interaction, *F*(12,5212) = 2.25, *p* = .008, *η*^2^_*p*_ = .01, 95% CI [.0004, .01]. Within-convoy comparisons identified age differences in tactics, specifically only looking within the inner, middle, and outer circles. Games-Howell pairwise comparison tests revealed that older adults had a significantly lower percentage of Neg Down tactics in their inner circle than younger adults and middle-aged adults (all *p’s* < .03; see Table 4 for means and Fig. 3 for within inner convoy comparison).^4^ Considering age differences are the main focus of the study and we found this age difference specifically within the most frequently used tactics of the convoy, we will only focus on these top tactics when analyzing the effectiveness of tactic use.

### The Predictive Value of State and Person-Level Top Tactic Use and Age for Affective Experience

To answer our second research question of how the use of top tactics at the level of regulation instances predicts self-reported post-regulation affect and whether this is moderated by age, we conducted a multilevel regression analysis using restricted maximum likelihood estimation (REML) method in R (R Core Team, 2021). These models evaluated self-reported pre- and post-regulation affect as a function of age and the use of top tactics in general and within three of the four categories outlined above (Pos up, Neg Down, and Acceptance; see page 14).

We used a two-level design. The levels are defined as repeated ESM occasions at level 1, nested within individuals at level 2. First, we fitted an unconditional model and calculated ICCs, all of which indicated sufficient variance in between-person level (42%) for two-level analysis. Within-person variables were calculated as occasion-specific and separate from the person means. Middle-aged adults were the reference group, such that younger and older-aged adults were included in the model for comparison. The burst variable was also centered to the first burst.

We tested for heterogeneity in multi-level models by comparing heterogeneous variance components to the homogeneous unconditional model. We defined a possible heterogeneity source as age group for between-levels. The model comparisons showed that none of them improved the models significantly. Hence, we did not include any variance heterogeneity parameter in the multilevel models (see Supplementary Materials for details).

We used a forward selection approach to add predictors to the models and compare across nested models using AIC and BIC information criteria and likelihood-ratio tests. We compared models to ensure that the best model fit indicated the lowest AIC and BIC values. Random slopes for within-person predictors (emotion regulation tactic categories) were also estimated, and likelihood ratio tests were conducted to determine whether allowing the slopes to vary across sessions improves model fit or not. Those that improved the model fit were kept in the final model but removed if they were not at *p* < .10 (Nezlek, 2011). Lastly, we calculated the overall *R*^2^ for the entire model to determine the variance explained by each model.

First, we tested the effect of burst, age, and pre-regulation affect on post-regulation affect (Model 1). Second, we included person-level top tactic use in general and in categories (Model 2). We also tested the interaction effects between age groups and top tactic categories to evaluate whether the effectiveness of top tactic use in categories (relationship to improved affect) changes across age groups (Model 3). We only kept those interactions that were significant and random effects that improved the model fit in the final model. Table 5 summarizes multilevel regression results for self-reported post-regulation affect.

**Table 5:** Multilevel models examining self-reported affect as a function of top tactic category use and age

	Model 1		Model 2		Model 3		Final model	
Predictors	*Est*	*SE*	*Est*	*SE*	*Est*	*SE*	*Est*	*SE*
(Intercept)	2.83^***^	.17	2.69^***^	.18	2.73^***^	.21	2.67^***^	.23
Burst	−.07^***^	.02	−.06^***^	.02	−.06^***^	.02	−.07^***^	.02
YA	.13	.10	.18	.10	.10	.19	.06	.22
OA	.37^***^	.10	.28^**^	.10	.27	.19	.53^***^	.16
Between-person predictors								
Self-reported pre-regulation affect	.09^*^	.04	.08^*^	.03	.08^*^	.04	.08^*^	.04
Top tactic use (overall)			−.04	.04	−.04	.05	−.06	.05
Top tactic categories								
Pos Up			.04	.05	.04	.05	.01	.05
Neg Down			−.10^*^	.05	−.09	.05	−.11^*^	.05
Acceptance			.00	.05	.01	.05	−.02	.06
Within-person predictors								
Self-reported pre-regulation affect	.46^***^	.01	.44^***^	.01	.44^***^	.01	.43^***^	.02
Top tactic use (overall)			.07	.08	−.19	.15	−.07	.15
Top tactic categories								
Pos Up			.41^***^	.04	.41^***^	.06	.36^***^	.04
Neg Down			−.13^***^	.04	−.06	.07	−.03	.06
Acceptance			−.07	.04	.18^*^	.07	.20	.12
OA * top tactic use (overall)					.30	.20		
OA * Pos Up					−.01	.09		
OA * Neg Down					−.01	.09		
OA * Acceptance					−.34^***^	.10	−.26	.14
YA * Top tactic use (overall)					.43^*^	.19	.31	.24
YA * Pos Up					.00	.08		
YA * Neg Down					−.18^*^	.09	−.23^*^	.09
YA * Acceptance					−.38^***^	.10	−.21	.17
Random effects								
Residual (L1)	1.05		.76		.75		.62	
Intercept (L2)	.75		.26		.26		.33	
Self-reported pre-regulation affect							.06	
Top tactic use (overall)							.90	
Neg Down Acceptance							.09 .51	
Marginal *R*^2^/Conditional *R*^2^	.016/.424		.445/.586		.447/.590		.406/.679	

*N* = 219, number of observations = 5324. *YA* younger adults, *MA* middle-aged adults, *OA* older adults. *L2*, level 2 (individual), *L1*, level 1 (instances). * *p* < .05 ** *p* < .01 *** *p* < .001

#### Self-Reported Post-Regulation Affect

Age explained 2.5% of the variance in between-person level post-regulation affect. Age differences were significant in the final model—older adults reported significantly higher post regulation affect than middle-aged and younger adults (*p* < .001).

Pre-regulation self-reported affect accounted for 26% of the within-level variance, and at the person-level accounted for 61% of the between-level variance of post-regulation affect. In both cases, pre-regulation affect was a significant predictor of post-regulation self-reported affect (*p*’s < .05).

Using a top tactic in general (regardless of category) did not significantly predict post-regulation affect, either at the between or within level. Among person-level tactic category items, the use of specifically negativity-downregulating as a top tactic significantly predicted lower self-reported affect. All other person-level tactic categories were not significant predictors. Person-level tactic category items explained 16% of between-person variance in self-reported post-regulation affect beyond age and pre-regulation affect.

At the within-level, the use of positivity-upregulating as a top tactic significantly predicted higher self-reported affect. Overall, top tactic category items explained 3% of the within-level variance beyond pre-regulation affect.

**Fig. 4:**
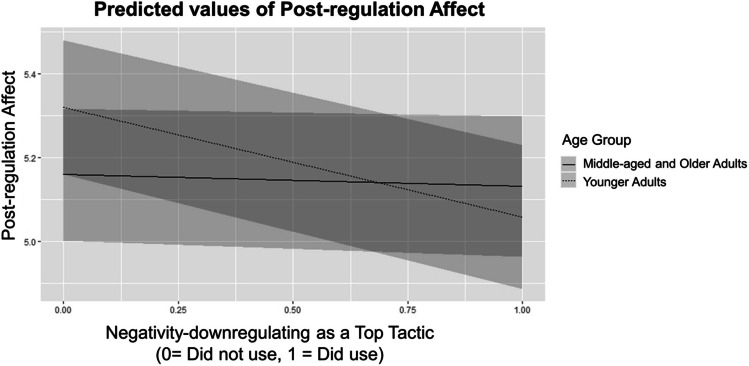
Younger adult × Neg Down top tactic interaction (depicts age group comparisons according to the middle-age group as a reference group)

We also tested the interaction effects between age and top tactic categories to understand whether emotion regulation tactic effectiveness varied among age groups. The only significant interaction observed was between negativity-downregulating and younger adults. Younger adults’ post-regulation affect was more negatively affected by using negativity-downregulating as a top tactic than the other age groups (see Fig. 4). Although acceptance was frequently used as a top tactic, it did not predict effectiveness and this relationship was also not moderated by age.

## Discussion

The current study examined age differences in emotion regulation tactic use in everyday life through novel *emotion regulation convoys.* Convoys provide snapshots of the hierarchical configurations of tactics an individual uses and allow us to determine what tactics individuals use relatively more than others. We investigated age differences in the make-up and effectiveness of the most frequently used tactics in convoys.

While prior work has suggested that age differences in emotion regulation might not account for age differences in emotional well-being (Isaacowitz, 2022) because age groups seem so similar to each other on the level of strategies, the emotion regulation convoy findings provide a closer parallel between emotion regulation behaviors and outcomes in aging. The convoy approach revealed age differences in relative tactic use for the most frequently used tactics; older adults use less negativity-downregulating as their top tactic than younger and middle-aged adults. Effectiveness findings revealed that the use of positivity-upregulating as a top tactic predicted higher post-regulation affect, but age did not moderate this finding. Using negativity-downregulating as a top tactic more negatively affected younger adults’ post-regulation affect. Considering younger adults use negativity-downregulating as a top tactic significantly more than older adults and younger adults’ mood is also more negatively affected by these tactics, our results suggest that younger adults’ convoys reflect relatively less effective tactics. Interestingly, younger adults are also more likely to believe other negativity-downregulating tactics such as cognitive and attention distraction are the most effective (Livingstone et al., 2020).

If older adults drop some of the less effective tactics from their repertoire of top tactics (negativity-downregulating) and rely to a greater extent on more effective ones (positivity-upregulating), this could be one way shifts in emotion regulation behavior could underlie age-related differences in emotional experience. Our findings, therefore, represent an important step in aligning adult age differences in emotion regulation behavior with adult age differences in affective experience.

### Limitations and Future Directions

Despite its innovative approach to mapping out individual differences in emotion regulation behavior at a point in time, this study does have some limitations. First, we did not assess all valence-based tactics to keep surveys short—it may be necessary for future work to include tactics that downregulate positivity to be comprehensive. Second, since there was no significant variation at the burst level, our study design of 3 one-month intervals was insufficient to see within-person changes. Despite this, researchers may still be interested in studying tactics in short time periods in future work; other contextual factors might cause individuals to experience shifts in emotion regulation and subsequent affect, such as during changing seasons.

Nonetheless, our findings suggest that we may be able to use convoys to observe possible within-person changes over time in the movement or stability of emotion regulation strategies. A critical future direction will therefore be to test for within-person changes in emotion regulation convoys over extended periods. This future longitudinal work can assess tactic movement across convoy circles over time: for example, what tactics get *promoted* by moving to a closer circle or *demoted* to a lower circle due to less frequent use. Are the cross-sectional age differences we observe due to older adults’ inner circle becoming more positive within these individuals because negativity-downregulating tactics get demoted while positivity-upregulating ones get promoted? Testing whether convoy make-up changes longitudinally will be a robust test of actual within-person change in emotion regulation, providing a holistic rather than piecemeal approach. It is also important to note that convoy makeup may remain fairly stable over extended periods, and the convoy approach can help identify that pattern as well.

### From Description to Theory

Descriptive work using emotion regulation convoys is a necessary precursor to testing theories of age-related changes in emotional processes; how can we test underlying mechanisms when we do not yet know the specific nature of within-person age-related changes? Several prominent conceptual models of emotional aging make claims about why emotion regulation may change with age, such as limited time perspective (Socioemotional Selectivity Theory; Carstensen et al., 1999), changes in physiological arousal (Strength and Vulnerability Integration; Charles, 2010), and underlying resources (Selection, Optimization, and Compensation in Emotion Regulation; Urry & Gross, 2010). Descriptive work may constrain which mechanisms are even plausible to consider. When findings suggest no age differences in strategies, it is hard to link to theories of age differences, so our cross-sectional findings in which older adults appear to use more effective tactics may be an important descriptive step toward eventually testing theories of age-related changes.

Certain situations may make some tactics more likely to use than others; if convoys change over time in concert with changes in situations individuals face, that could provide an alternative mechanism for age differences. Linking convoys to specific situations may also improve measures of emotion regulation variability (Benson et al., 2019; Eldesouky & English, 2018) by providing more precise information about the particular tactics different group members switch between in relation to specific situational factors.

Emotion regulation convoys provide a holistic, hierarchical snapshot of emotion regulation behavior. These cross-sectional findings suggest age differences in convoys in a potentially adaptive direction, with older adults’ convoys featuring more effective tactics than younger adults. Longitudinal work is needed to test within-person changes in convoys over time, which can then be considered with regard to key theories of emotional aging.

We believe that emotion regulation convoys can also be useful for testing potential group differences or intraindividual changes in emotion regulation behavior beyond aging: for example, cultural differences or changes during psychotherapy. In all cases, we cannot test underlying mechanisms without a clear picture of the hierarchical configuration of emotion regulation behavior at a particular time, and this is what emotion regulation convoys provide.

### Supplementary Information

Below is the link to the electronic supplementary material.

Supplementary file1 (DOCX 30 KB)

## Footnotes

1 Our original pre-registered analyses stated that we would exclude surveys that were less than 80% completed. However, upon further examination, this resulted in a significant data loss. Therefore, in order to maintain more instances in the analyses, we opted to keep all participants, resulting in the lowest number of instances being 13 in a single burst.

2 This was a slight deviation from our pre-registration which focused on six tendency scores (3 valence-based and 3 strategy-based) as the main analyses, derived mainly from frequency sums.

3 All further analyses that use convoys (age differences and multi-level models) were specified as exploratory in our pre-registration because this was our first attempt to create and test convoys.

4 Between convoy comparisons specifically compared the percentage of tactics in inner, middle, and outer circles to each other, looking within age groups. Younger adults had significantly higher percentages of Pos Up in their middle circle compared to all of their other circles, where middle-aged and older adults had significantly lower percentages of Pos Up in their outer circle than their other circles (all* p*’s < .02; see Table 4 for all means and Fig. 2 for convoy depictions aggregated across bursts).

## Additional Information

### Data Availability

De-identified data and analysis code are posted on the OSF repository at this private link: https://osf.io/wv9m3/?view_only=a63c101e90154395983c5da4d7fda3c7. All other study materials can be obtained by emailing the corresponding author.
