# Supplementary material for: Emotion Regulation Convoys: Individual and Age Differences in the Hierarchical Configuration of Emotion Regulation Behaviors in Everyday Life
Source: Affect Sci. 2023 Dec 16;4(4):630–43. doi: 10.1007/s42761-023-00228-8 (PMC10751281; doi:10.1007/s42761-023-00228-8)
Supplement: Supplementary file 1 — Supplementary file1 (DOCX 30 KB) [file 42761_2023_228_MOESM1_ESM.docx]

**Emotion regulation convoys: Individual and age differences in the hierarchical configuration of emotion regulation behaviors in everyday life**

**Marissa A. DiGirolamo, Shevaun D. Neupert, and Derek M. Isaacowitz**

**Author Note**

*Affective Science* submission.

Correspondence concerning this article should be addressed to Marissa A. DiGirolamo, Department of Psychology, Northeastern University. Email: m.digirolamo@northeastern.edu

**Supplementary Materials**

**Checking Variance Heterogeneity in Multilevel Models**

Regression models require the error variances to be equal for the different subpopulations, known as variance homogeneity assumption. We ran homogenous and heterogenous models to check this assumption in a multilevel setting and evaluated whether heterogeneous variance components improved models significantly. In model comparisons, we used AIC, BIC information criteria, as well as LR likelihood ratios. In model comparisons, lower values of AIC and BIC indicate better fit. In the LR ratio test, a significant difference between the two models suggests that the model with more parameters (in our case, heterogenous model) fits better.

We defined possible heterogeneity sources as age groups for between levels and tested variance heterogeneity for the dependent variable of self-reported post regulation affect. All model comparisons resulted in no significant LR ratio test and lowered AIC and BIC values for homogenous models.

**Table 1**

*All other measures included in the study, split by Recruitment, Intake, Phone Survey, Life Events Survey, and Debrief.*

| **Measure** | | **# of Items** | **Source** | | **Description** | | | |  |
| --- | --- | --- | --- | --- | --- | --- | --- | --- | --- |
| **Recruitment** | |  |  | |  | | | |  |
| Telephone Mini-Mental Status Exam | | 9 | Adapted from Newkirk, et al., 2004 | | Checking for qualification to participate in study (no cognitive impairment) | | | |  |
| **Intake** | |  |  | |  | | | |  |
| Demographics | | 11 |  | | Gender, ethnicity, race, age, education level, religion, native/non-native English speaker, general health, employment, marital status | | | |  |
| Positive and Negative Affect Schedule | | 2 (20) | Watson & Tellegen, 1988 | | Measures trait-level emotion | | | |  |
| Satisfaction with Life Scale | | 6 | Diener et al., 1985 | | Measures cognitive judgement of life satisfaction | | | |  |
| Center for Epidemiologic Studies Depression Scale | | 2 (20) | Radloff, 1977 | | Measures depressive symptoms | | | |  |
| Emotional Maturity Scale | | 14 | Adapted from Singh & Bhargava, 1991 | | Emotional maturity | | | |  |
| Emotional Self-Efficacy Scale | | 6 | Adapted from Kirk et al., 2008 | | Self-report emotional intelligence; four subscales measure ability to understand, perceive , facilitate and regulate emotions | | | |  |
| Emotional Regulation Questionnaire | | 11 | Gross & John, 2003 | | Measure cognitive reappraisal and expressive suppression tendencies | | | |  |
| Savoring Beliefs Inventory | | 25 | Bryant, 2003 | | Assess percieved ability to derive pleasure from past/present/future positive events | | | |  |
| Trait Meta-Mood Scale | | 31 | Salovey et al., 1995 | | Measure individual differences in monitoring,  evaluating, and regulating feelings and emotions | | | |  |
| Overall Preferences | | 16 |  | | Self-report emotion regulation tactic use | | | |  |
| Effectiveness | | 16 |  | | Self-report effectiveness of ER tactics | | | |  |
| **Experience Sampling (mEMA)** | |  |  | |  | | | |  |
| AffectNow | | 1 |  | | Current affect | | | |  |
| Regulate | | 1 |  | | Yes/No tried to regulate emotions | | | |  |
|  | |  |  | |  | | | |  |
| Event/Goal/Context | | 3 |  | | Event where ER was attempted | | | |  |
| SitSel | | 2 |  | | Yes/No *select a situation* | | | |  |
| SitMod | | 3 |  | | Yes/No *take action* | | | |  |
| Att | | 2 |  | | Yes/No *shift attention* | | | |  |
| Rea | | 3 |  | | Yes/No *reattribute* | | | |  |
| Expression | | 2 |  | | Yes/No *change expression* | | | |  |
| RegOther | | 2 |  | | Yes/No other | | | |  |
| Affect Before/After | | 2 |  | | Affect before/after event | | | |  |
| S8I | | 9 |  | | Situational Eight DIAMONDS | | | |  |
| AnythingElse | | 2 |  | | Add additional info | | | |  |
|  | |  |  | |  | | | |  |
| StrongEmo | | 1 |  | | Yes/No experience emotions | | | |  |
| Doing/Interacting | | 3 |  | | Current situation | | | |  |
| SitSelPeople | | 3 |  | | Yes/No *avoid/seek people* | | | |  |
| SitSelActivity | | 3 |  | | Yes/No *avoid/seek activity* | | | |  |
|  | |  |  | |  | | | |  |
|  | |  |  | |  | | | |  |
| **Major Life Events Survey (mEMA)** | |  |  | |  | | | |  |
| Describe most important event | | 3 |  | |  | | | |  |
| Event Characteristics Questionnaire | | 18 |  | |  | | | |  |
| Affect before/during/after event | | 5 |  | |  | | | |  |
| **Debrief** | |  |  | |  | | | |  |
| Shipley Vocabulary Test | | 21 |  | | | | Assess crystalized intelligence | |  |
| Wechsler Adult Intelligence Scale - Revised | | 16 | Weschler, 1955 | | | | Assess working memory capacity |  |  |
| Letter Fluency (phonemic) | 2 | | | Spreen & Benton, 1977 | | Assess phonemic verbal fluency | | |  |
| Animal Naming (categorical) | 1 | | | Rosen, 1980 | | Assess categorial verbal fluency | | |  |
|  |  | | |  | |  | | |  |
| Big Five Inventory | 2 (62) | | | Adapted from Goldberg, 1993 | | Big Five personality test | | |  |
| Revised Life Orientation Test | 11 | | | Adapted from Scheier, 1994 | | Optimism vs. pessimism about the future | | |  |
| Midlife Development Inventory | 12 | | | Adapted from Lachman & Weaver, 1997 | | Measure perceived control | | |  |
| Physiology | 17 | | |  | | Background to help interpret physiological responses | | |  |
| Emotional Maturity Scale | 14 | | | Adapted from Singh and Bhargava, 1991 | |  | | |  |
| Overall Preferences | 17 | | |  | |  | | |  |
| Effectiveness | 16 | | |  | |  | | |  |

**Table 2**

*Fixed-Effects ANOVA results using Percentage as the criterion as a function of a 4 (Categories: Positivity Upregulating, Negativity Upregulating, Negativity Downregulating, Acceptance) x 3 (Age Group: Young - YA, Middle - MA, Older - OA) x 3 (Circle: Inner, Middle, Outer) x 3 (Burst: 1,2,3) design*

| Predictor | Sum  of  Squares | *df* | Mean  Square | *F* | *p* | _partial_ η^2^ | _partial_ η^2^  95% CI  [LL, UL] |
| --- | --- | --- | --- | --- | --- | --- | --- |
| (Intercept) | 220.30 | 1 | 220.30 | 4249.40 | .000 |  |  |
| Age Group | 0.00 | 2 | 0.00 | 0.01 | .992 | .00 | [.00, 1.00] |
| Burst | 0.01 | 2 | 0.00 | 0.10 | .903 | .00 | [.00, .00] |
| Category | 21.23 | 3 | 7.08 | 136.53 | .000 | .07 | [.06, .09] |
| Circle | 0.04 | 2 | 0.02 | 0.38 | .683 | .00 | [.00, .00] |
| Age Group x Burst | 0.03 | 4 | 0.01 | 0.12 | .974 | .00 | [.00, 1.00] |
| Age Group x Category | 0.31 | 6 | 0.05 | 0.99 | .430 | .00 | [.00, .00] |
| Burst x Category | 0.28 | 6 | 0.05 | 0.92 | .482 | .00 | [.00, .00] |
| Age Group x Circle | 0.13 | 4 | 0.03 | 0.64 | .631 | .00 | [.00, .00] |
| Burst x Circle | 0.00 | 4 | 0.00 | 0.02 | .999 | .00 | [.00, 1.00] |
| Category x Circle | 51.99 | 6 | 8.67 | 167.16 | .000 | .16 | [.14, .18] |
| Age Group x Burst x Category | 0.31 | 12 | 0.03 | 0.49 | .920 | .00 | [.00, .00] |
| Age Group x Burst x Circle | 0.06 | 8 | 0.01 | 0.15 | .996 | .00 | [.00, 1.00] |
| Age Group x Category x Circle | 1.40 | 12 | 0.12 | 2.25 | .008 | .01 | [.0004, .01] |
| Burst x Category x Circle | 0.58 | 12 | 0.05 | 0.94 | .505 | .00 | [.00, .00] |
| Age Group x Burst x Category x Circle | 1.54 | 24 | 0.06 | 1.24 | .192 | .01 | [.00, .01] |
| Error | 270.20 | 5212 | 0.05 |  |  |  |  |

*Note.* LL and UL represent the lower-limit and upper-limit of the partial η^2^ confidence interval, respectively.
